# The Cancer Observatory of Cali, a priority

**Published:** 2018-09-30

**Authors:** Claudia Patricia Abreu, María Fernanda Tobar Blandon, Jorge Alirio Holguín

**Affiliations:** 1 Grupo Cáncer, Secretaria de Salud Pública Municipal. Cali, Colombia; 2 Facultad de Salud, Universidad del Valle. Cali, Colombia

In the last issue of Colombia Medica, the Cali Population Registry of Cancer (RPCC, for its initials in Spanish) documented that important advances in cancer control have been achieved. Mortality due to this group of diseases has decreased significantly during the last 25 years, thanks to the partial control of cancers related to infectious agents (stomach and cervix) and cancers associated with tobacco consumption (lung, bladder, oral cavity). However, there is less control of cancers related to screening activities (breast, prostate and colorectal), and hematolymphoid tumors that have successful treatment options with chemotherapy. As a result, cancer survival in children and adults is 20 percentage points below that observed in Europe and the United States ^1^.

One strategy to improve the indicators is the creation of a cancer observatory in the city. Health observatories are conceived as smart, proactive, dynamic, synchronic and participatory systems that generate knowledge for the formulation of policies and strategies that allow to impact, in a positive way, the social determinants of health ^2^. With this perspective and in congruence with the technical line of health observatories given by the Ministry of Health and Social Protection, it is necessary to generate strategies; to have articulation spaces; and to integrate sources of information, which in addition to being standardized and synchronized, facilitate the monitoring and identification of trends in the behavior of events, and the understanding and explanation of the health situation of the population based on complex analyses of inequalities and health vulnerabilities ^2^.

In order to complement the analysis of the cancer situation in Cali, it is a priority to implement the Cali Cancer Observatory for monitoring the interventions made in the city, by integrating the information from the RPCC with the information on risk management ^3^, the notification of cancer to the public health system and the administrative records that the involved participants (such as health service providers, insurers, suppliers of drugs and other technologies that impact cancer care) periodically produce about care, costs and results. There is a need to coordinate efforts intended to strengthen the generation of knowledge in other aspects related to the diagnostic opportunity, risk groups, interventions, their effectiveness, their benefit, the costs for the system and patients, and the perception of the population in relation to their achievement; among other issues of interest around cancer.

The Cali Cancer Observatory may become the central axis of knowledge management through surveillance, research, analysis and information on the health situation regarding cancer. It can be an effective tool to monitor the impact of the Ten-Year Plan for cancer control in Cali; and for evaluating the results of the implementation of the C/Can 2025 Initiative ^4^. Cali was the first city in the world to implement the ***C/Can 2025: City Cancer Challenge*** , which is committed to supporting the city with the aim of improving the health of our population and reducing inequalities in access to a quality oncological care, through a network of committed multi-sectorial partners, such as the Ministry of Social Protection, the National Cancer Institute, the Municipal Public Health Secretariat of Cali, the Secretariat of Health of the Province of Valle de Cauca, the Cancer Population Registry of Cali at Universidad del Valle, the Insurers, Health Service Providers, NGOs, scientific societies, inspection, monitoring and control agencies, and civil society; as well as agencies such as the UN and WHO, and other national and international agencies linked to this issue. 

Cali is the city of reference for health care for the population of southwestern Colombia. The interventions that are made in the city can benefit the population of its zone of influence, which represents 25% of the 46 million inhabitants of Colombia. Patients come to Cali looking for diagnosis and treatment of oncological diseases, in health institutions of medium and high complexity. No health institution in Cali has the installed capacity to handle the 9,000 new cases of cancer per year diagnosed in the city's oncology services network ^1^. Therefore, a multi-sectorial work is needed, in order to improve the results obtained to date.

Given these challenges and aware of the implications of cancer in population health, the municipal administration has prioritized this group of diseases as of interest in the public agenda, not only at local level but also in national and international scenarios. This multi-sectorial effort is fundamental to increase coverage and improve the quality of oncological care by intervening in four areas: basic oncology services; management of oncological services; quality of cancer care; and community's access to integrated care.

The Municipality of Cali has identified as strategic the creation of the Cali Cancer Observatory for the generation of knowledge around cancer; intended to recognize, analyze and describe the behavior of cancer beyond morbidity and mortality; so that it be constituted in a knowledge management laboratory, for the formulation of political, cultural, social and economic interventions for a well-known problem.

The Secretariat of Municipal Health of Cali, with the epidemiological surveillance group and the accompaniment of the Universidad del Valle, has been making important efforts to establish, with more precision, the cancer situation in the city; for which it is based on the information of the RPCC, which is considered as the most important source of descriptive epidemiology of cancer in Latin America (http://rpcc.univalle.edu.co/es/index.php).

All these aspects are of great importance to address the opportunities that allow improving risk management, based on innovation in clinical management and administrative management for comprehensive cancer care in Cali. This supports the decision of the Municipal Public Health Secretariat, in conjunction with the RPCC, to create the Cali Cancer Observatory, from a new vision of public health that integrates other levels of analysis, and to understand the impact of the determinants of health in the process of life, care, health-disease process; so that the main function of public health be engaged in the orientation of policies and strategies that favor the improvement of living conditions of people.
