# Supplementary material for: The Cancer Observatory of Cali, a priority
Source: Colomb Med (Cali). 2018 Sep 30;49(3):249–50. (PMC6220482)
Supplement: Related texts

# Letter: El Observatorio de Cáncer de Cali, una prioridad

El último número de Colombia Médica el Registro Poblacional de Cáncer de Cali (RPCC) (https://www.ncbi.nlm.nih.gov/pubmed/29983461) documentó que se han logrado importantes avances en el control del cáncer. La mortalidad por este grupo de enfermedades ha disminuido de manera significativa durante los últimos 25 años gracias al control parcial de los canceres relacionados con agentes infecciosos (estómago y cuello uterino) y los cánceres asociados con el consumo del tabaco (pulmón, vejiga, cavidad oral). Sin embargo, hay menos control de los cánceres relacionados con actividades de tamización (mama, próstata y colorrectal) y en los tumores hematolinfoides que tienen opciones de tratamiento exitoso con quimioterapia. Como resultado, la supervivencia al cáncer en niños y adultos se encuentra 20 puntos porcentuales por debajo de la observada en Europa y Estados Unidos ^1^.

Una estrategia para mejorar los indicadores es la conformación de un observatorio del cáncer en la ciudad. Los observatorios de salud, se conciben como sistemas inteligentes, proactivos, dinámicos, sincrónicos y participativos que generan conocimiento para la formulación de políticas y estrategias que permiten impactar de manera positiva los determinantes sociales de la salud ^2^. Con esta perspectiva y en congruencia con la línea técnica de los observatorios en salud dada por el Ministerio de Salud y Protección Social, se requiere generar estrategias, disponer de espacios de articulación, integrar las fuentes de información, que además de estar estandarizadas y sincronizadas, faciliten el monitoreo y la identificación de tendencias en el comportamiento de los eventos, la comprensión y explicación de la situación de salud de la población a partir de análisis complejos de las desigualdades, las inequidades y las vulnerabilidades en salud ^2^. 

Para complementar el análisis de la situación del Cáncer en Cali, es prioritario implementar el Observatorio de Cáncer de Cali para el monitoreo de las intervenciones que se hagan en la ciudad, integrando la información del RPCC con la información de la gestión del riesgo ^3^, la notificación de cáncer al sistema de salud pública y los registros administrativos que los actores del sistema producen periódicamente sobre atenciones, costos y resultados. Existe la necesidad de articular esfuerzos para fortalecer la generación de conocimiento en otros aspectos relacionados con la oportunidad diagnóstica, los grupos de riesgo, las intervenciones, su efectividad, el beneficio, los costos para el sistema y los pacientes y la percepción de la población frente al logro de las mismas; entre otros asuntos de interés alrededor del Cáncer.

El Observatorio de Cáncer de Cali puede constituirse en el eje central de la gestión del conocimiento a través de la vigilancia, la investigación, el análisis y la información de la situación de salud respecto al cáncer. Puede ser una herramienta eficaz para monitorear el impacto del Plan decenal para el control del cáncer en Cali y para evaluar los resultados de la implementación de la Iniciativa C/Can 2025 ^4^. Cali fue la primera ciudad en el mundo en implementar la **Iniciativa C/Can 2025: Desafio de Ciudades Contra el Cáncer**, que tiene el compromiso de apoyar a la ciudad con el objetivo de mejorar la salud de nuestra población y reducir las desigualdades en el acceso a una atención oncológica de calidad, a través de una red de socios multisectoriales comprometidos, tales como el Ministerio de la Protección Social, el Instituto Nacional de Cancerología, la Secretaria de Salud Pública Municipal de Cali, la Secretaria de Salud del Departamento del Valle de Cauca, el Registro Poblacional de Cáncer de Cali de la Universidad del Valle, las Aseguradores, los Prestadores de servicios de salud, ONGs, las sociedades científicas, las instancias de inspección, vigilancia y control y la sociedad civil, además de agencias como la ONU y la OMS y otras agencias nacionales e internacionales vinculadas a este tema. 

Cali es la ciudad referente de la atención en salud para la población del suroccidente colombiano. Las intervenciones que se hagan en la ciudad pueden beneficiar a la población de su zona de influencia que representa el 25% de los 46 millones de habitantes de Colombia. A Cali acuden los pacientes para el diagnóstico y tratamiento de las enfermedades oncológicas, en las instituciones de salud de mediana y alta complejidad. Ninguna institución sanitaria de Cali tiene capacidad instalada para atender los 9,000 casos nuevos anuales de cáncer que son diagnosticados en la red de servicios oncológicos de la ciudad ^1^. Por lo tanto se necesita de un trabajo multisectorial para lograr mejorar los resultados obtenidos hasta la fecha. 

Ante estos retos y conscientes de las implicaciones del Cáncer en la salud poblacional, la administración municipal ha priorizado este grupo de enfermedades como de interés en la agenda pública, no sólo en el ámbito local sino también en los escenarios nacionales e internacionales. Este esfuerzo multisectorial es fundamental para aumentar la cobertura y mejorar la calidad de la atención oncológica interviniendo cuatro áreas: los servicios oncológicos básicos; la gestión de los servicios oncológicos; la calidad de la atención del cáncer y el acceso de la comunidad a una atención integrada. 

El Municipio de Cali ha identificado como estratégico la creación del Observatorio de Cáncer de Cali para la generación de conocimiento alrededor del Cáncer; para reconocer, analizar y describir el comportamiento del Cáncer más allá de la morbilidad y la mortalidad; de forma que se constituya en un laboratorio de gestión del conocimiento, para la formulación de Intervenciones de orden político, cultural, social y económico frente a una problemática estudiada. 

La Secretaria de Salud Municipal de Cali, con el grupo de vigilancia epidemiológica y el acompañamiento de la Universidad del Valle, viene realizando importantes esfuerzos para establecer con mayor precisión, la situación del cáncer en la ciudad; para la cual se apoya en la información del RPCC considerado como la fuente de epidemiología descriptiva de cáncer más importante de Latinoamérica (http://rpcc.univalle.edu.co/es/index.php) . 

Todos estos aspectos son de gran importancia para abordar las oportunidades que permitan mejorar la gestión del riesgo, a partir de la innovación en la gestión clínica y la gestión administrativa para la atención integral del Cáncer en Cali. Lo anterior sustenta la decisión de la Secretaria de Salud Pública Municipal en conjunto con el RPCC de crear un Observatorio de Cáncer de Cali, desde una nueva visión de salud pública que integre otros niveles de análisis y que permita comprender el impacto de los determinantes de la salud en el proceso de vida, cuidado, proceso salud-enfermedad; para que la función principal de la salud pública se comprometa en la orientación de las políticas y estrategias que favorezcan mejorar las condiciones de vida de las personas
